# Complement factor 5 blockade reduces porcine myocardial infarction size and improves immediate cardiac function

**DOI:** 10.1007/s00395-017-0610-9

**Published:** 2017-03-03

**Authors:** Soeren E. Pischke, A. Gustavsen, H. L. Orrem, K. H. Egge, F. Courivaud, H. Fontenelle, A. Despont, A. K. Bongoni, R. Rieben, T. I. Tønnessen, M. A. Nunn, H. Scott, H. Skulstad, A. Barratt-Due, T. E. Mollnes

**Affiliations:** 1 Department of Immunology, Oslo University Hospital, Rikshospitalet, P.b. 4950 Nydalen, 0424 Oslo, Norway; 2 K.G. Jebsen IRC, University of Oslo, Oslo, Norway; 3 Intervention Centre, Oslo University Hospital, Oslo, Norway; 4 Division of Emergencies and Critical Care, Department of Anaesthesiology, Oslo University Hospital, Oslo, Norway; 5 Immunology Research Centre, St. Vincent’s Hospital, Melbourne, VIC Australia; 6 Department of Clinical Research, University of Bern, Bern, Switzerland; 7 Akari Therapeutics Plc, London, UK; 8 Department of Pathology, Oslo University Hospital, University of Oslo, Oslo, Norway; 9 Department of Cardiology, Oslo University Hospital, Rikshospitalet, University of Oslo, Oslo, Norway; 10 Research Laboratory, Nordland Hospital, Bodø, Norway; 11 Faculty of Health Sciences, K.G. Jebsen TREC, University of Tromsø, Tromsø, Norway; 12 Centre of Molecular Inflammation Research, Norwegian University of Science and Technology, Trondheim, Norway

**Keywords:** Ischemia/reperfusion, Myocardial infarction, Complement, C5, Contractility, LTB4

## Abstract

Inhibition of complement factor 5 (C5) reduced myocardial infarction in animal studies, while no benefit was found in clinical studies. Due to lack of cross-reactivity of clinically used C5 antibodies, different inhibitors were used in animal and clinical studies. Coversin (*Ornithodoros moubata* complement inhibitor, OmCI) blocks C5 cleavage and binds leukotriene B4 in humans and pigs. We hypothesized that inhibition of C5 before reperfusion will decrease infarct size and improve ventricular function in a porcine model of myocardial infarction. In pigs (*Sus scrofa*), the left anterior descending coronary artery was occluded (40 min) and reperfused (240 min). Coversin or placebo was infused 20 min after occlusion and throughout reperfusion in 16 blindly randomized pigs. Coversin significantly reduced myocardial infarction in the area at risk by 39% (*p* = 0.03, triphenyl tetrazolium chloride staining) and by 19% (*p* = 0.02) using magnetic resonance imaging. The methods correlated significantly (*R* = 0.92, *p* < 0.01). Tissue Doppler echocardiography showed increased systolic displacement (31%, *p* < 0.01) and increased systolic velocity (29%, *p* = 0.01) in coversin treated pigs. Interleukin-1β in myocardial microdialysis fluid was significantly reduced (31%, *p* < 0.05) and tissue E-selectin expression was significantly reduced (*p* = 0.01) in the non-infarcted area at risk by coversin treatment. Coversin ablated plasma C5 activation throughout the reperfusion period and decreased myocardial C5b-9 deposition, while neither plasma nor myocardial LTB4 were significantly reduced. Coversin substantially reduced the size of infarction, improved ventricular function, and attenuated interleukin-1β and E-selectin in this porcine model by inhibiting C5. We conclude that inhibition of C5 in myocardial infarction should be reconsidered.

**Electronic supplementary material:**

The online version of this article (doi:10.1007/s00395-017-0610-9) contains supplementary material, which is available to authorized users.

## Introduction

The introduction of early reperfusion therapy of acute myocardial infarction (MI) in the clinical setting has decreased morbidity and mortality and improved post-MI cardiac function. However, a considerable part of the ischemic myocardium is still lost upon reperfusion. Ischemia and reperfusion cause liberation of damage associated molecular patterns (DAMP) from ischemic or injured cells, activating innate immune responses, a prerequisite for the healing process, currently reviewed in [19]. However, overactivation causes detrimental effects by injuring the myocardium, an effect termed ischemia/reperfusion injury (IRI) [22], leading to aggravated infarct size and pump failure.

Complement is an upstream sensor and effector system of innate immunity, a key system for immune surveillance and homeostasis, but also implicated to play a critical role in the pathophysiology of myocardial IRI [4, 35]. Complement as a danger sensing alarm system relies on soluble pattern recognition receptors of three different activation pathways, the classical, the lectin and the alternative pathway [35]. They all converge at the central component C3, which is cleaved into C3a and C3b and subsequently leads to cleavage of C5, which generates the potent anaphylatoxin C5a and the terminal C5b-9 complement complex, both exerting proinflammatory effector functions [35].

Complement inhibition in myocardial infarction was first shown to reduce infarction size in rodents already in 1990 [47]. Experimental studies investigating complement inhibition in a clinically relevant context are rare, i.e. the inhibitor was given after onset of ischemia, but confirmed the protective potential of C5 inhibition [44]. Pigs are highly recognized for the translational value of results obtained [20], however C5 inhibition has not been tested as no inhibitors for pig C5 have been available. Inhibition of various other parts of the complement cascade by inhibition of complement factor 1 [21], treatment with soluble complement receptor 1 [2], protecting the endothelium with dextran sulfate [3] and tyrosine-O-sulfate [4] clearly showed the potential of complement inhibition in pigs. Clinical studies with the C5-antibody pexelizumab were therefore performed without prior preclinical testing and the results were disappointing [15, 31]. Administration of the anti-C5 antibody during percutaneous coronary intervention neither reduced myocardial infarction nor decreased mortality [23]. However, a major concern with these studies was that complement activation measured by soluble C5b-9 (sC5b-9), the final activation product that should be completely blocked by the antibody, increased similarly in the treatment and the placebo groups [31] leading to discussion whether a too low dose of the anti-C5 drug had been used.

The tick derived, specific C5 inhibitor coversin (*Ornithodoros moubata* Complement Inhibitor, OmCI), prevents equally efficiently the cleavage of C5 in humans and pigs [6, 32]. The potency of coversin in inhibiting C5 in comparison to the clinically used C5 inhibitor eculizumab, which has been derived from the same clone as its predecessor pexelizumab [43], is not known. Additionally, coversin also has an internal binding pocket for leukotriene B4 (LTB4) [39], an arachidonic acid metabolite thought to play a role in myocardial IRI [25]. However, the magnitude and effect of LTB4 binding on the physiologic effects of coversin are uncertain.

We hypothesized that the C5 inhibitor coversin could reduce infarct size and improve myocardial function in a clinically relevant porcine model of acute myocardial infarction.

## Materials and methods

### Animal preparation

The ethics committee of the Norwegian Food Safety Authority approved this study in pigs (approval number: 68/11-3811) and all experiments were performed in concordance with the guidelines from Directive 2010/63/EU of the European Parliament on the protection of animals used for scientific purposes. Housekeeping, anesthesia, euthanasia, and recording of hemodynamic and respiratory parameters were performed in accordance to ARRIVE guidelines as shown in table (Online Resource 1) and as reported previously [5]. Briefly, anesthesia was induced in twenty-one 20 kg pigs by intramuscular ketamine (800 mg), azaperone (80 mg), atropine (1 mg) followed by intravenous (iv) pentobarbital 1–3 mg kg^−1^ and maintained using iv morphine 1–2 mg kg^−1^ h^−1^ and isoflurane 1.0–1.5% in oxygen/air mixture. After sternotomy, a silastic occluding tape was placed around the left anterior descending (LAD) coronary artery distal to the second diagonal branch allowing reversible complete occlusion. Microdialysis catheters (CMA 71, 100 kDa cut-off, 2 cm membrane, 1 µl min^−1^ flow, M Dialysis, Solna, Sweden) were placed in the LAD dependent area and in a control region supported by the left circumflex artery (Cx).

### Experimental protocol

Ischemia was induced for a total of 40 min by LAD occlusion, except for sham animals. Twenty minutes prior to reperfusion, sixteen animals were randomized to treatment with coversin or saline (NaCl 0.9%, placebo group), *n* = 8 in each group. Coversin (Akari Therapeutics Plc, London, UK) has a plasma half-life of about 30 h due to stable binding to C5 [18] and was diluted in saline. It was given as a 1 mg kg^−1^ bolus, and followed by a continuous infusion of 0.036 mg kg^−1^ h^−1^ [5]. The control group and the three sham animals received the same amount of saline without coversin. Fifteen minutes before euthanasia, iv magnetic resonance imaging (MRI) contrast agent gadoteric acid (0.4 mM kg^−1^, Dotarem, Guerbet, Paris, France) was given [34]. Just before euthanasia, LAD was re-occluded and iv Evans Blue (2% in 40 ml phosphate buffered saline, Sigma Aldrich, St. Louis, MO, USA) was given to delineate the area at risk (AAR). Euthanasia was carried out by iv injection of pentobarbital (500 mg), morphine (30 mg), and potassium chloride (50 mmol). After euthanasia, the heart was excised and rinsed in ice-cold saline.

Arterial blood samples were obtained prior to surgery, after stabilization prior to induction of ischemia, at the end of 40 min of ischemia, and every hour throughout the reperfusion period. Samples were taken for blood gas analysis, serum, and EDTA-plasma preparation and were immediately cooled and centrifuged prior to storage at −80 °C. Microdialysis samples and thermal dilution cardiac output were obtained at the same time points. After euthanization, tissue samples were taken from the center of the Evans blue free area (AAR), at the border of the Evans blue free area (border zone) and in the Evans blue stained Cx region (control area) and snap-frozen in approximately 1 ml OCT™ (Sakura Finetek Europe, Zoeterwoude, the Netherlands) prior storage at −80 °C.

### Infarct size assessed by magnetic resonance imaging

After tissue sampling, air-filled balloons were placed in the left and right ventricle. MRI analysis was performed using a 3 Tesla scanner (Philips, the Netherlands). T1-weighted images (3D FFE, TR/TE = 5.4/2.3 ms, flip angle 35°, BW = 434 Hz, 125 slices and scan duration = 02:15) with a measured isotropic resolution of 0.8 mm covering the entire heart were acquired using a quadrature head coil. Additionally, T1 measurement sequence was performed (Look Locker sequence: T1w TFE with “shared” inversion pulse, TR/TE = 2.3/4.3 ms, flip angle = 3°, inversion delay = 38.4 ms, phase interval = 65.5 ms, BW = 853 Hz, SENSE factor 2, isotropic resolution of 1 mm), and T1 maps were reconstructed using NordicIce (NordicNeuroLab, Bergen, Norway). The segmentation of the infarcted volumes was done in OsiriX [37]. T1map was used to discriminate infarcted areas with the 3D region-growing tool (threshold of 400). The used threshold lead to inclusion of pericardium and endocardium as well but as the amount is comparable and small in all groups and subjective manual processing would have been necessary, we did not subtract it from the total infarcted volume. Infarction size (ml) was determined in T1 weighted images and compared to the total left ventricular volume.

### Infarct size assessed by histological staining

After MRI, the left ventricle was cut in 5 mm thick slices. The non-stained AAR was dissected and immersed in tetrazolium chloride (TTC, 1% in phosphate buffered saline, Sigma Aldrich, St. Louis, MO, USA) at 38°C for 20 min. Slices were placed in 4% formaldehyde solution (Histolab Products AB, Gothenburg, Sweden) on ice for 30 min prior to digital scanning. Infarct size was determined as percentage of AAR as described previously [20] using Photoshop CS5 (Adobe Systems Software Ltd., Ireland).

### Echocardiography

Systolic left ventricular function was assessed by echocardiography from a four-chamber view prior to ischemia and at the end of the reperfusion period (GE Vivid 7, Horton, Norway). Peak systolic velocity and systolic displacement of the mitral plane were obtained from pulse Doppler echocardiography and averaged from the septum and the lateral wall (Echopac PC Version 112, GE Vingmed Ultrasound, Horten, Norway).

### Immunofluorescence analysis

The snap-frozen tissues were cut into 5 μm thick sections, air-dried for 60 min and fixed with cold acetone for 10 min. They were either processed immediately or stored at −80 °C until further analysis. Then, after hydration, the sections were stained using a two-step indirect immunofluorescence technique. For E-selectin, the following primary and secondary antibodies were used: mouse anti-human E-selectin (Sigma, St. Louis, MO, USA) and goat anti-mouse IgG-Alexa546 (Molecular probes, Carlsbad, CA, USA). The antibodies used for Fibrinogen-like protein 2 (FGL-2) were rabbit anti-FGL2 (Aviva Systems Biology Corp, San Diego, CA, USA) and sheep anti-rabbit IgG-Cy3 (Sigma, St. Louis, MO, USA). A nuclear staining was performed using 4′,6-diamidino-2-phenylindole (DAPI; Sigma, St. Louis, MO, USA). A fluorescence microscope (DMI4000B; Leica, Wetzlar, Germany) was used to analyze the slides and the quantification of fluorescence intensity was performed using Image J software, version 1.50 (https://rsb.info.nih.gov/ij/) on TIFF images. All pictures were taken under the same conditions to allow for correct quantifications and comparison of fluorescence intensities.

### In vitro assessment of complement inhibitory effects of coversin and eculizumab

Human and porcine whole blood samples anticoagulated with lepirudin (Celgene, Marburg, Germany) were pre-incubated with coversin or eculizumab (Alexion Pharmaceuticals, CT, USA) in a twofold serial dilution (final concentrations of 1.6, 0.8, 0.4, 0.2 and 0.1 µM) or PBS for the uninhibited control in sterile polypropylene tubes for 5 min at 37 °C. Subsequently, blood specimens were stimulated with zymosan at a final concentration of 50 µg/ml, or PBS for the negative control. After 30 min, the reaction was stopped by adding EDTA (final concentration 10 mM), samples centrifuged (3000*g*, 15 min, 4 °C). The resulting plasma was stored at −80 °C before analysis of C5b-9. Human and porcine serum samples were pre-incubated with coversin or eculizumab in a twofold serial dilution (final concentrations of 3.2, 1.6, 0.8, 0.4, 0.2 and 0.1 µM) or PBS for the uninhibited control in sterile polypropylene tubes for 5 min (room temperature) before analysis for functional complement activity.

### Functional complement activity and C5b-9 (TCC)

Commercially available enzyme immune assay (Complement System Screen Wieslab; Euro Diagnostica, Malmö, Sweden) and murine anti-human C5b-9 antibody (clone aE11, Dako, Glostrup, Denmark) were used according to manufacturer’s instructions to detect functional complement activity and sC5b-9 production in plasma, respectively. Both methods detect the respective human and pig epitopes [41]. In tissue, the membrane form of C5b-9 was visualized in frozen sections from the AAR, border zone and control area. Tissue samples were incubated for 30 min at room temperature using the murine anti-human C5b-9 antibody (clone aE11, Dako, Glostrup, Denmark) diluted 1/25 in Dako antibody diluent (Dako K8006, Glostrup, Denmark), washed in phosphate buffered saline and stained by Ventana ultra View Universal DAB Detection Kit (Ventana Medical Systems, Inc., Tucson, AZ) according to the manufacturer’s instructions. A Nikon Eclipse E1000M microscope was used and photos were obtained with original 40× magnification.

### Myocardial metabolism and inflammation

Microdialysis fluid from the AAR and control Cx region and EDTA-plasma was assessed for inflammatory mediators interleukin (IL)-1β, IL-6, IL-8, IL-10, and TNF using a porcine multiplex cytokine assay on a Bio-Plex 100 system (Bio-Rad, Hercules, CA, USA) as previously described [9]. LTB4 from plasma and myocardial tissue was measured using a competitive enzyme immunoassay according to the manufacturer’s instructions (R&D systems, Minnesota, MN, USA).

### Markers of cardiac injury

Serum troponin-T levels were determined at the institutional clinical laboratory (Modular E170, Roche Diagnostics, Switzerland). Plasma heart fatty acid binding protein H-FABP levels were measured by ELISA in accordance to manufacture’s instruction (Hycult Biotech, Uden, The Netherlands).

### Statistics

Investigators were blinded to the treatment during the experiments and all analyses.

Two animals died immediately after reperfusion due to ventricular fibrillation (one coversin and one placebo treated animal) and were excluded. Thus, functional complement activity was analyzed in 16 animals subjected to LAD occlusion and three sham-operated animals. Complete inhibition of all three complement pathways by coversin treatment was confirmed in all animals, except for one, which was excluded after statistical confirmation of outlier behavior (Grubbs’ test, *p* < 0.05). Thus, 15 animals (seven coversin and eight control animals) were used in all further analyses if not stated otherwise.

Two animals (one coversin and one control animal) had significantly smaller AAR determined by Evans Blue staining due to anatomical variations of the LAD and were therefore excluded from MRI analysis. Microdialysis catheters ceased function before 120 min of reperfusion in two coversin and one control animal and statistical comparison was therefore done with five and seven animals, respectively.

If not stated otherwise, values are presented as mean ± standard deviation (SD). Values obtained for coversin treated and control animals were compared at defined time points using Mann–Whitney *U* test. Two-way ANOVA was used if more than two groups had to be compared. Linear mixed effect model (intervention as fixed effect and subject number as random effect) was used to compare groups throughout the whole study period. Multiple comparisons were post hoc Bonferroni corrected. The Pearson correlation coefficient was calculated to compare infarct sizes determined by TTC and MRI. Statistical analyses were performed using SPSS 22 (IBM, Armonk, NY, USA) and GraphPad Prism 6 (GraphPad Software, La Jolla, CA, USA).

## Results

### Effect of coversin on myocardial infarction size

#### Evaluation by histological staining

**Fig. 1:**
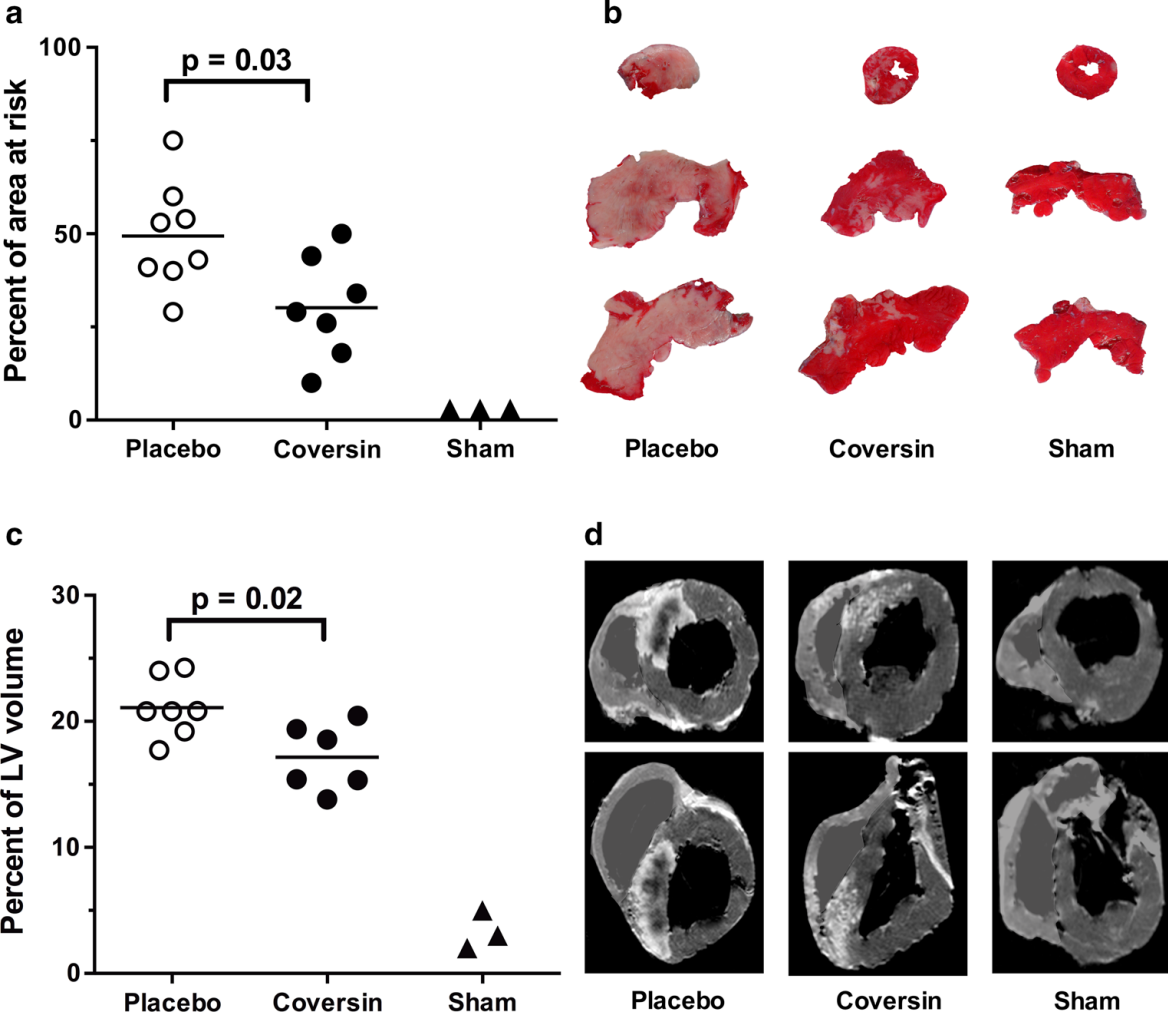
Coversin reduced infarction size. **a** Coversin (C5 inhibitor) reduced infarction in the area at risk (AAR) by 39%, *p* = 0.03 determined by TTC staining. **b** TTC staining of the AAR (example slices from one animal in each group) shows infarcted areas in white and non-infarcted areas in *red*. **c** Coversin reduced infarction in the left ventricle by 19%, *p* = 0.02 determined by gadolinium stained magnetic resonance imaging (MRI). **d** Transversal (*first row*) and frontal (*second row*) T1-weighted MRI images of the same animals shown in panel B with shaded right ventricle as only the left ventricle was analyzed. *White area* and *black area* within white area depict infarction and non-perfused infarction, respectively. *Horizontal line* denotes mean [*n* = 8 (placebo) and *n* = 7 (coversin)]. Mann–Whitney *U* test. *LV* left ventricle

Myocardial ischemia and reperfusion led to an average infarct size of 49.4 ± 14.2% (mean ± SD, necrotic tissue as % of the AAR) in the control group. Coversin treated animals showed an infarct size of 30.1 ± 14.0% of the AAR, representing a significant reduction of 39% as compared to controls (*p* = 0.03, Fig. 1a, b). The AAR was comparable between coversin treated and control animals as determined by Evans Blue staining (21.2 ± 6.4 and 25.5 ± 5.5% of left ventricular volume, respectively; *p* = 0.12, data not shown).

#### Evaluation by post mortem MRI

Infarcted volume in the left ventricle was decreased from 21.1 ± 2.4% in placebo treated animals to 17.2 ± 2.7% in coversin treated animals as determined by MRI (19% reduction, *p* = 0.02, Fig. 1c, d). Infarction determined by TTC staining and magnetic resonance imaging were highly correlated (*R* = 0.92, *p* < 0.01, Online Resource 2).

Sham-operated animals, in which the LAD was not ligated, did not reveal any signs of myocardial ischemia nor infarction evaluated by histological staining and MRI. Also in all other analysis reported in this study, sham treated animals were consistently stable at baseline levels throughout the study period and are therefore not reported in further results.

### Effect of coversin on myocardial function

**Fig. 2:**
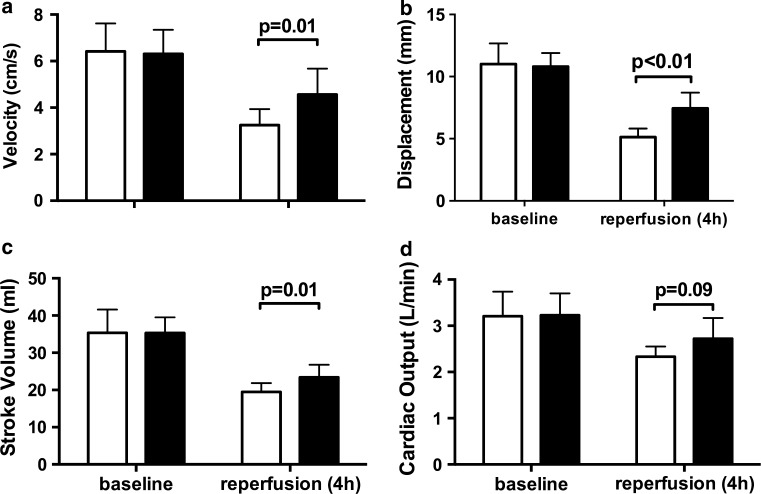
Coversin improved myocardial function. Tissue Doppler echocardiography was evaluated from the mitral plane and averaged from septal and lateral wall movements. *Open bars* represent control and *filled bars* coversin treated animals. Systolic velocity was reduced at 4 h after reperfusion in both groups but was 29%, *p* = 0.01 higher in coversin compared to control animals (**a**). Likewise, systolic displacement was 31%, *p* < 0.01 higher in coversin treated animals in comparison to placebo treated animals (**b**). Thermal dilution derived stroke volume (**c**) was 14%, *p* = 0.01 higher, while cardiac output (**d**) showed a trend of 16%, *p* = 0.09 increase in coversin treated animals. Values presented as mean ± SD [*n* = 8 (placebo) and *n* = 7 (coversin)]. Mann–Whitney *U* test

Myocardial function was measured by tissue Doppler echocardiography, whereas cardiac output and stroke volume were measured by thermal dilution at start and end of the experiment (Fig. 2). Peak systolic velocity was 29% higher in the coversin treated animals than in the controls (4.6 ± 1.1 and 3.3 ± 0.7 cm s^−1^, respectively; *p* = 0.01, Fig. 2a). Likewise, systolic displacement was 31% higher in coversin treated animals than in controls (7.4 ± 1.3 and 5.1 ± 0.7 mm, respectively; *p* < 0.01, Fig. 2b). Stroke volume was 16% higher in the coversin treated animals than in the controls (23.4 ± 3.4 and 19.5 ± 2.4 ml, respectively; *p* = 0.01, Fig. 2c). Cardiac output showed a non-significant trend to higher values in coversin treated animals compared to the controls (2.7 ± 0.4 and 2.3 ± 0.2 l/min, respectively; *p* = 0.09, Fig. 2d).

### Effect of coversin on local myocardial inflammation

#### Microdialysis

**Fig. 3:**
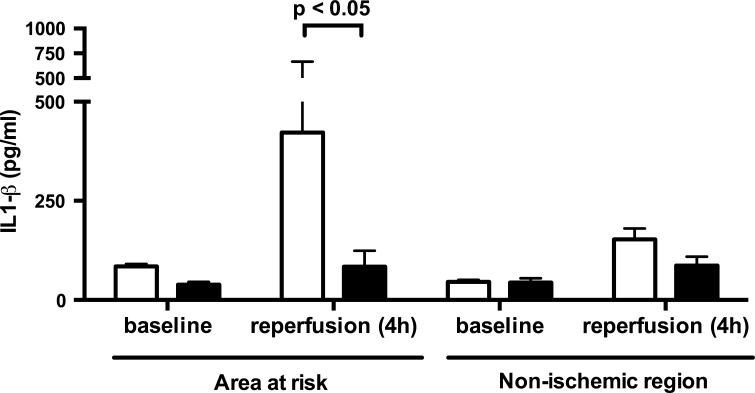
Coversin reduced local myocardial IL-1β production. IL-1β obtained by microdialysis was induced in the area at risk (AAR) and not the control region after 4 h of reperfusion. Coversin treatment (*filled bars*) significantly reduced IL-1β in the AAR by 80% in comparison to placebo treated animals (*open bars*). Values presented as mean ± SEM [*n* = 7 (placebo) and *n* = 5 (coversin)]. Two-way ANOVA with post hoc Bonferroni correction for multiple testing

The inflammasome-related IL-1β was increased at the end of reperfusion in the AAR only and this increase was significantly blunted by coversin treatment (Fig. 3). IL-6 and IL-8 increased during reperfusion, both without significant effect of coversin treatment, while IL-10 and TNF did not increase form baseline levels (data not shown).

#### Immunofluorescence

**Fig. 4:**
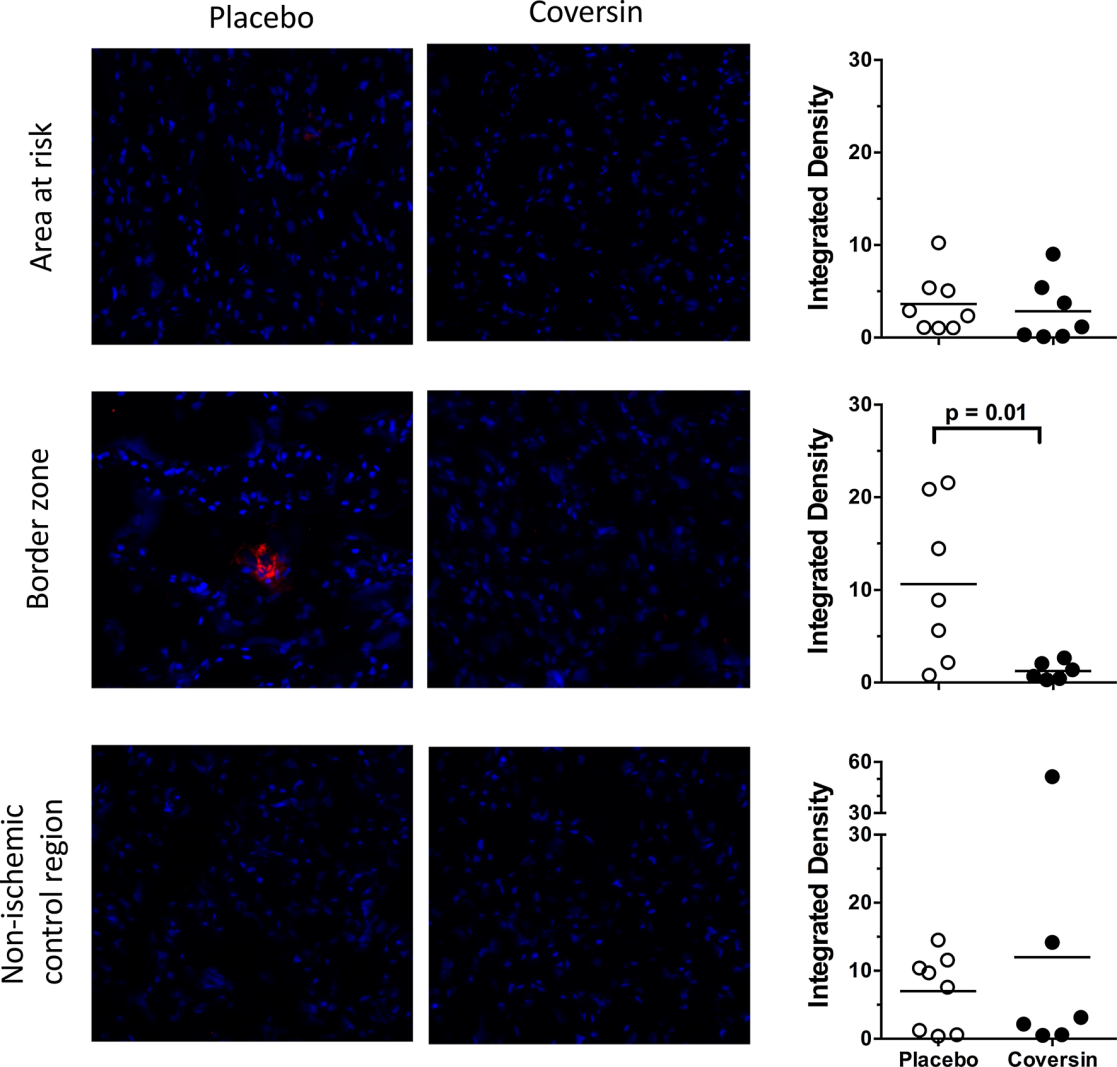
Coversin reduced E-selectin expression. Myocardium was stained with antibody against E-selectin. E-selectin expression was increased in placebo treated animals in the border zone of the AAR and unchanged in the center of the AAR and non-ischemic Cx control region (*left panels*). Coversin treatment led to significant decrease of E-selectin, expressed by reduced density of staining (*middle* and *right panels*). *Horizontal line* denotes mean [*n* = 8 (placebo) and *n* = 7 (coversin)]. Mann–Whitney *U* test

In control animals, myocardial ischemia and reperfusion led to increased expression of E-selectin in the border zone of the AAR, while E-selectin in both the infarcted center of the AAR and Cx control region was not changed (Fig. 4, left panels). Coversin significantly reduced the E-Selectin expression in the border zone (Fig. 4, middle and right panels). FGL-2 was increased in the infarcted center of the AAR and the Cx control region in comparison to sham treated animals without a significant effect of coversin (data not shown).

### Systemic and local myocardial effect of coversin on complement and LTB4

**Fig. 5:**
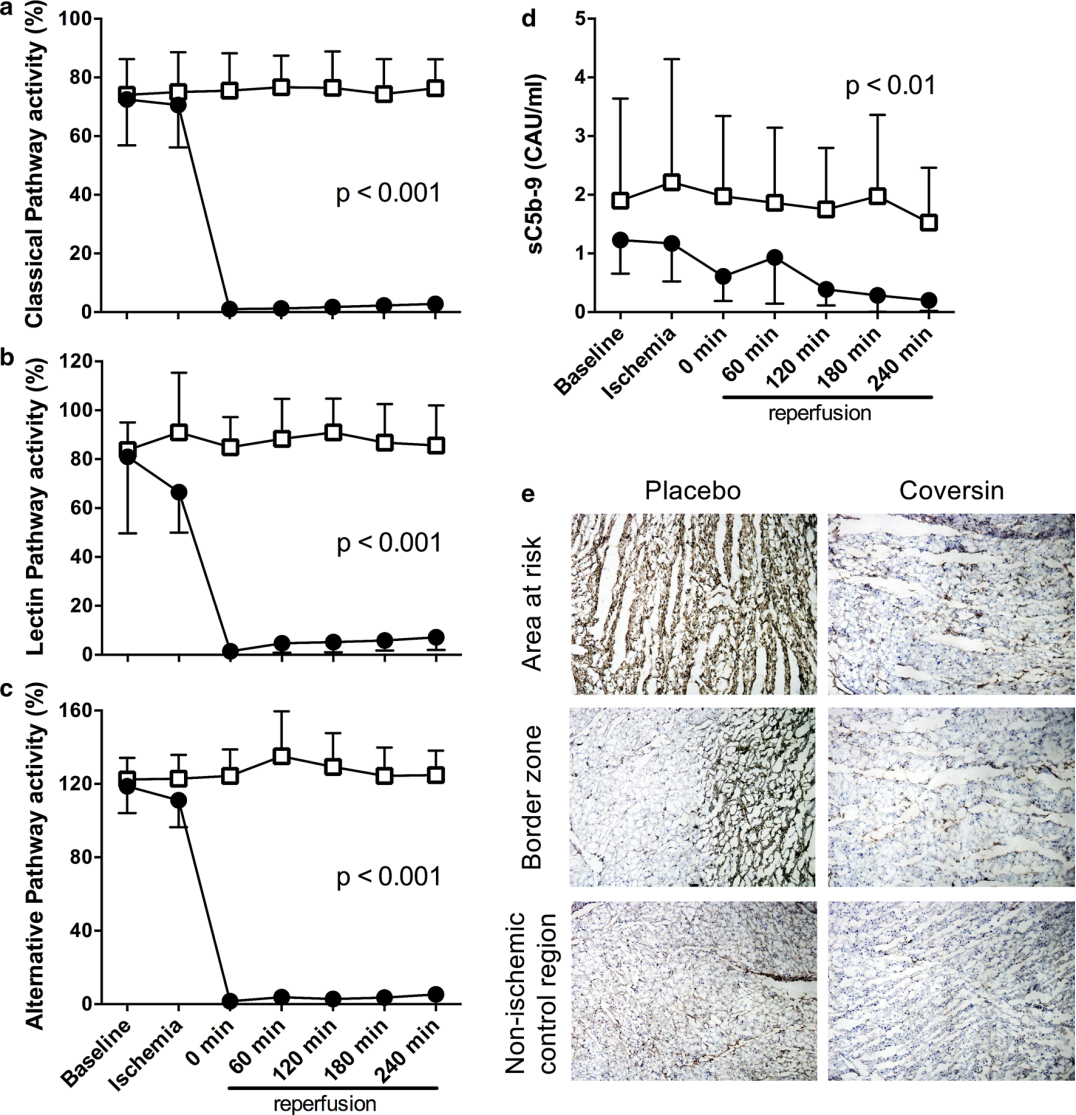
Coversin eliminated complement activity. Complement activity was assessed in plasma and the classical (**a**), lectin (**b**) and alternative pathway (**c**) were monitored using C5b-9 deposition as common readout. Coversin bolus treatment during coronary ischemia led to significantly reduced complement activity in all pathways (*filled circles*) and was not affected in control animals (*open boxes*). Complement activity remained low in all three pathways throughout the reperfusion period until the end of the experiment. Consequently, the plasma soluble complement activation product sC5b-9 was significantly reduced in plasma of coversin treated animals in comparison to controls (**d**). Myocardium was stained with an antibody against C5b-9 (**e**). Visually, deposition of C5b-9 (*brown*) was markedly decreased in the area at risk, the border zone and the non-ischemic control region in coversin treated animals in comparison to placebo treated animals. **a**–**d** Values presented as mean ± SD [*n* = 8 (placebo) and *n* = 7 (coversin)]. Linear mixed effect model. *CAU* complement arbitrary units. **e** Results of two representative animals are shown

Complement activity was measured at all time points throughout the experiment. Coversin completely ablated complement activity measured via all the three complement activation pathways throughout the reperfusion period, whereas the activity remained unchanged in the placebo group (Fig. 5a–c). Coversin treatment significantly reduced sC5b-9 to levels below baseline, in contrast to the placebo group and consistent with complete inhibition of terminal complement (*p* < 0.01, Fig. 5d). Dense deposition of the C5b-9 complex in placebo treated animals was observed in the AAR, in the border zone, and to a lesser extent in the non-ischemic control region (Fig. 5e, left panels). Coversin treatment almost completely prevented C5b-9 deposition in AAR, the border zone, and non-ischemic control region (Fig. 5e, right panels).

**Fig. 6:**
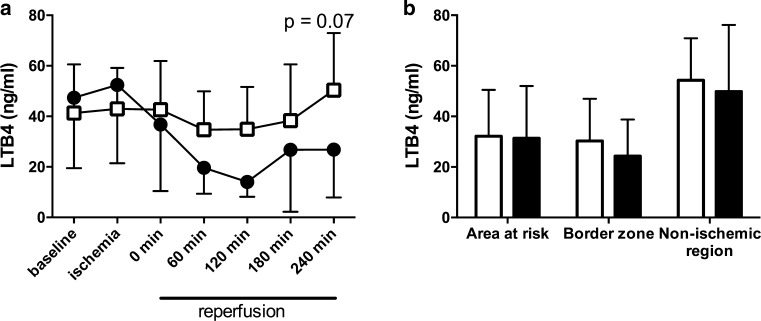
Coversin did not reduce LTB4. **a** LTB4 was assessed in plasma throughout the study period. LTB4 showed a non-significant trend to lower values during reperfusion in coversin treated animals compared to placebo. LTB4 in myocardial tissue from three different regions at the end of the experiment was not affected by Coversin treatment (*filled bars*) in comparison to control (*open bars*) (all *p* > 0.1). Values presented as mean ± SD. **a** Linear mixed effect model, **b** two-way ANOVA with post hoc Bonferroni correction for multiple testing

Plasma LTB4 concentrations during reperfusion were lower in coversin treated animals but not significantly different from placebo (*p* = 0.07, Fig. 6a). Myocardial LTB4 concentration was not affected by treatment in AAR, border zone, nor non-ischemic control region (Fig. 6b).

### Systemic effect of coversin as assessed by plasma analyses

**Fig. 7:**
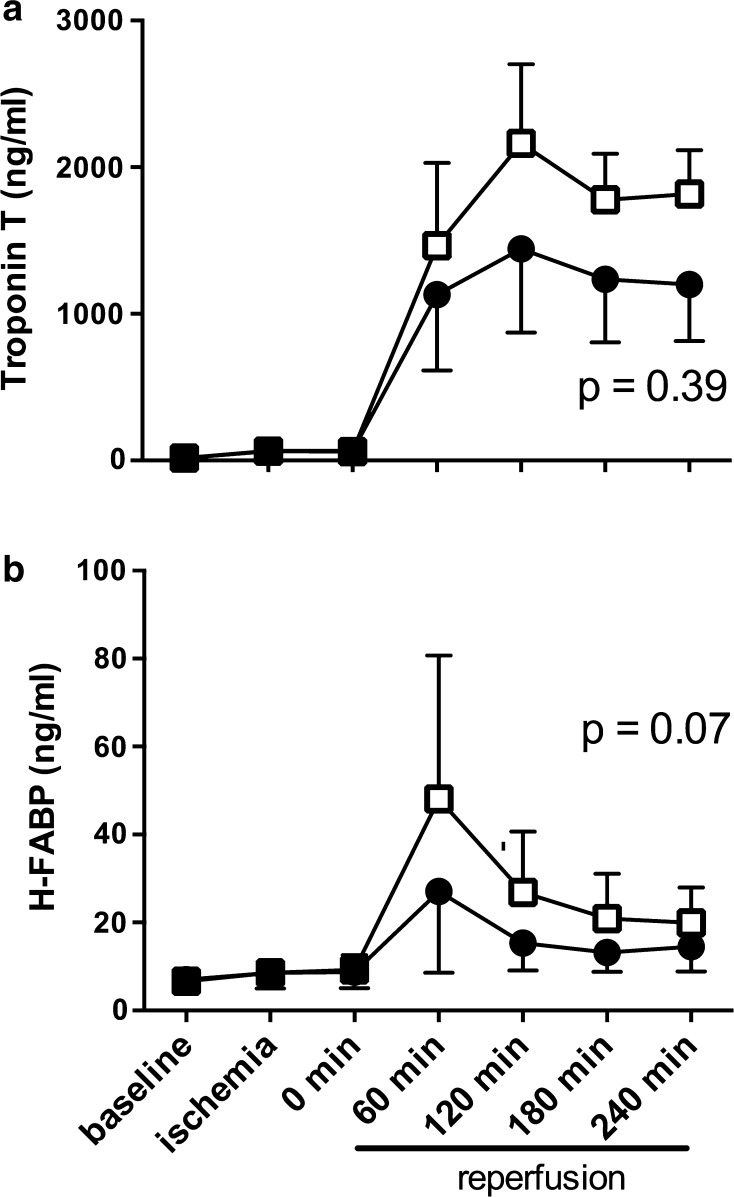
Coversin did not significantly decrease plasma markers of myocardial ischemia. Troponin T (**a**) and H-FABP (**b**) were detected in plasma throughout the study period. Myocardial ischemia lead to an increase in troponin T and H-FABP in both control (*open boxes*) and coversin treated (*filled circles*). Coversin treated animals showed a trend towards lower H-FABP levels throughout the whole reperfusion period without reaching significance in comparison to control animals (troponin T: *p* = 0.39; H-FABP: *p* = 0.07). Values presented as mean ± SEM [*n* = 8 (placebo) and *n* = 7 (coversin)]. Linear mixed effect model

Plasma troponin T and H-FABP increased in both the placebo and coversin groups during the reperfusion period confirming myocardial cell damage during the ischemic event (Fig. 7). Lower troponin T and H-FABP values were obtained in coversin treated animals in comparison to control animals throughout the reperfusion period without reaching significance, though a trend for lower values was observed for H-FABP (*p* = 0.07, Fig. 6b).

Plasma concentrations of IL-1β, IL-6, IL-8, IL-10 and TNF remained at baseline levels throughout the study period (data not shown).

### Comparison of coversin and eculizumab on complement activation

**Fig. 8:**
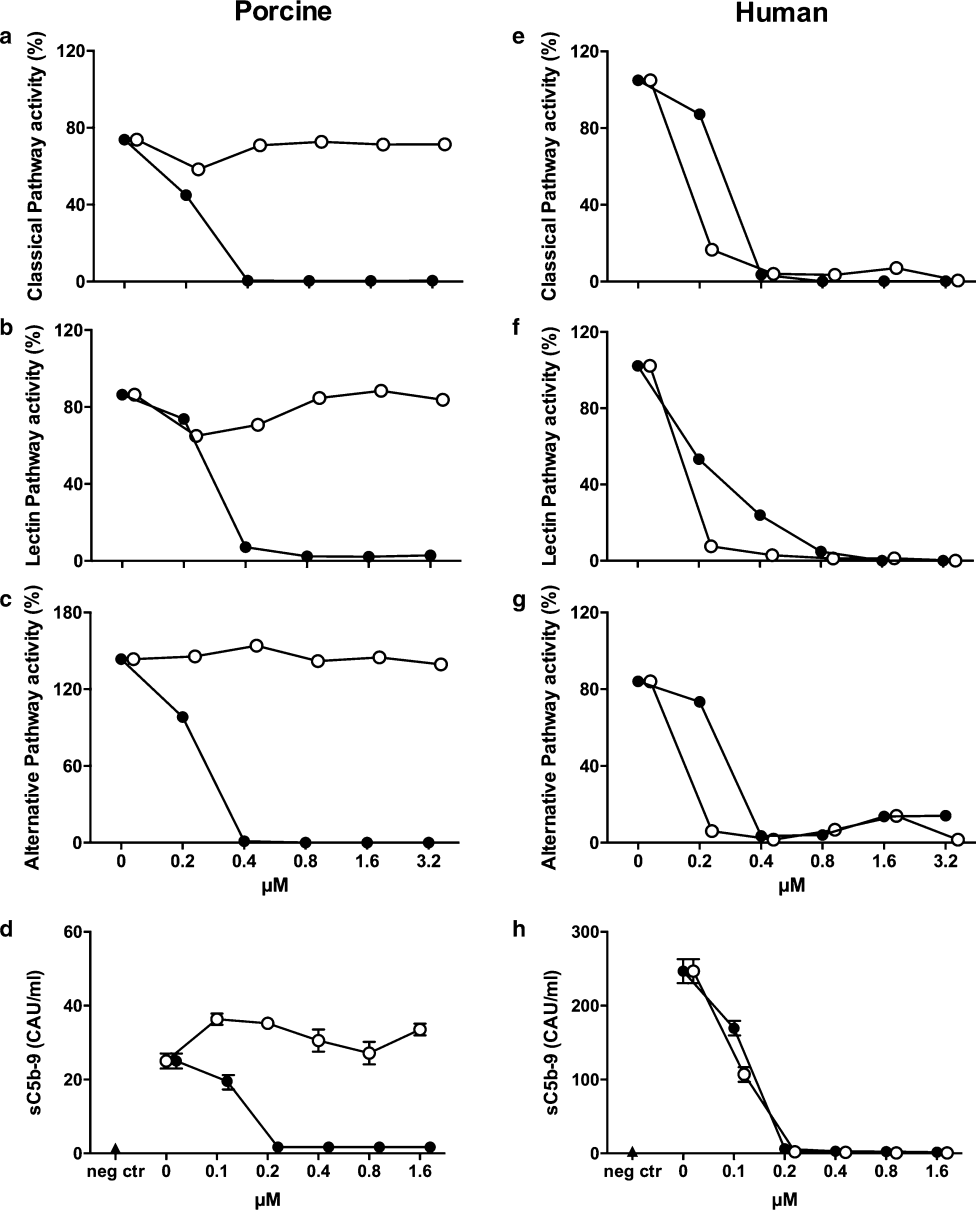
Coversin, but not eculizumab, inhibits porcine complement activation. Complement inhibitory effect of coversin (*filled circles*) and eculizumab (*open circles*) were assessed in the functional classical (**a**, **e**), lectin (**b**, **f**), and alternative pathway (**c**, **g**) assays in porcine (**a**–**c**) and human (**e**–**g**) serum using percentage of solid phase C5b-9 deposition as readout. Porcine (**d**) and human (**h**) whole blood was incubated with the complement activator zymosan and the effect of the inhibitors was examined using the soluble sC5b-9 complex as readout. Coversin, but not eculizumab, effectively inhibited porcine complement activity in a dose dependent manner, and was effective at the calculated in vivo concentration of 0.8 µM used in this study. Human complement activity was effectively inhibited by both inhibitors in a dose dependent manner. Complement activity of all three pathways was analyzed in duplicates and plasma from zymosan activated whole blood samples was analyzed in triplicates. *CAU* complement arbitrary units, *neg ctr* negative control, *sC5b-9* soluble C5b-9

Coversin, but not eculizumab, effectively inhibited functional complement activity in porcine serum (Fig. 8a–c), while both were equally effective in human serum (Fig. 8e–g). Similarly, formation of the fluid phase sC5b-9 by the complement activator zymosan in porcine whole blood was efficiently inhibited by coversin, but not eculizumab (Fig. 8d). Both inhibitors were again equally effective in human whole blood where they completely prevented zymosan-induced sC5b-9 formation (Fig. 8h).

## Discussion

In this porcine study of myocardial IRI, C5 inhibition by coversin prior and during reperfusion significantly reduced infarct size and improved ventricular function. Complete blockade of terminal complement pathway by coversin was revealed by lack of systemic complement activity in plasma and abolished deposition of C5b-9, which was extensive in the AAR in the control group. Finally, IL-1β and E-Selectin expression in the AAR were significantly reduced by coversin.

Targeting the complement system at the terminal stage preventing C5 cleavage is a reasonable approach as proximal complement activity is left unaffected and thus important immunoprotective and immunoregulatory functions exerted particularly by C3 are preserved [12]. End products of complement activation are C5a and C5b-9. Membrane bound C5b-9 induces inflammatory responses in the course of IRI by platelet and endothelial cell activation accompanied by leukocyte infiltration [11]. The potent anaphylatoxin C5a is regarded as a crucial factor in myocardial IRI [4, 24]. In our study, the detrimental effects of C5 cleavage were prevented resulting in protective effect on both infarct size and myocardial function. It is noteworthy that comparable porcine studies where C5a effect was diminished by C5a receptor antagonism [45] or a C5a monoclonal antibody  [1] showed less protection of the AAR and no effect on ventricular function. This highlights the importance of C5b-9 in myocardial reperfusion injury, while improvement of ventricular function confirms the physiological relevance of our findings. However, specific effects of coversin on myocardial function need to be investigated in studies observing long-term effects after myocardial IRI.

Leukotrienes are important multifunctional mediators of inflammation and promote neutrophil chemotaxis and adherence to capillary walls [48]. LTB4 is expressed on leucocytes after myocardial IRI [36], gets elevated in plasma in the course of myocardial infarction [42] and has been shown to be able to discriminate between cardiac and non-cardiac chest pain [26]. Coversin has an internal binding pocket capturing LTB4 and C5-inhibition prevents LTB4 formation [5]. In the present study, LTB4 in plasma did not significantly increase in the course of ischemia nor during reperfusion in placebo treated animals. This may be related to the short reperfusion time of 4 h in this study, as a doubling of LTB4 in humans appears during the first 24 h after acute myocardial infarction, probably in the course of endothelial cell activation [42]. However, neither plasma nor myocardial LTB4 concentrations were affected by coversin treatment indicating a negligible effect of coversin on LTB4 in this model. Furthermore, selective LTB4 blockade has only exhibited minor effects on myocardial IRI in rodents [8]. These findings indicate that the main coversin related effects observed in this study could be attributed to C5 inhibition, while LTB4 inhibition might add to the effect of C5 inhibition in long-term studies.

Large clinical studies have explored the efficacy of C5 inhibition using pexelizumab, a monoclonal antibody blocking C5 cleavage, on the outcome of myocardial infarction treated with thrombolysis [30] and percutaneous coronary intervention [15, 23]. These studies did not demonstrate convincing beneficial effects and several questions have arisen in the aftermath. Firstly, the dosing regimen of pexelizumab was only tested once, yet this has been decisive for dosages in subsequent studies [15]. Secondly, complement activity was insufficiently inhibited in both studies, and blood samples from the last trial revealed a similar increase in the formation of sC5b-9 in both placebo and treatment group [31]. This supports the notion that full inhibition of C5 is necessary to effectively reduce the harmful effects of complement activity in the heart. Ideally, coversin should have been compared to the formerly used pexelizumab or today’s clinically used eculizumab, which all inhibit cleavage of C5 at different binding sites [27]. However, pexelizumab and eculizumab are monoclonal antibodies with specificity for human C5 only [10] and we have shown that they do not interact with porcine C5. In this study, 0.85 µM coversin was used. In the clinical trials, 1.2 µM pexelizumab was used [14], which is equivalent to 0.6 µM eculizumab because of the double-binding property of the antibody eculizumab in contrast to the single-chain variant pexelizumab [38]. Thus, slightly higher doses of inhibitors were used in this study compared to the clinical studies, which may explain the successful prevention of reperfusion injury in this study but more importantly add evidence to the assumption that the pexelizumab dose may have been too low in order to achieve full C5 inhibition. Thirdly, administration of the C5 inhibitor in the clinical studies was probably given too late, only minutes prior to reperfusion in the hospital [23]. Therapy aiming at reduction of myocardial reperfusion injury should be initiated as early as possible after diagnosis of ischemia [22]. In this study, we aimed to mimic the clinical situation and initiated coversin treatment with a considerable time-gap prior to reperfusion. This is comparable to the clinical situation when medical treatment is started at the time of diagnosis in the prehospital setting with a time-gap prior to interventional reperfusion therapy. This approach should be easily transferrable to clinical trials.

Coversin treatment abolished IL-1β induction, which is cleaved in the inflammasome from inactive proIL-1β and is regarded as an inducer of sterile inflammation in myocardial IRI [46]. Interestingly, C5 activation and membrane bound C5b-9 have been shown to directly activate the inflammasome [29, 33], suggesting that reduced cell death and significant reduction of IL-1β observed in the present study is related to C5-inhibition. E-Selectin is essential for leukocyte recruitment, is a good marker of endothelial cell activation and the expression is IL-1β dependent [33]. Thus, the observed reduction in E-selectin expression in the border zone of the AAR in the present study might be caused by C5 inhibition through IL-1β, explaining the reduced reperfusion injury. The lack of significant increase in the rest of the cytokines might be explained by the short reperfusion time, as generation of cytokines is time-dependent and additionally affected by the limited recovery in microdialysis [28].

Pigs do not possess coronary collaterals, while humans experiencing myocardial ischemia often do. To compensate for this limitation, we therefore adopted the length of the occlusion period in this study (40 min) to a comparable length of 4 h of infarction in man [17]. Isoflurane was used as anesthetic agent in this study, although the cardioprotective properties of isoflurane are known. We chose this gas as it confers myocardial stability. Both groups received identical amounts of isoflurane and the infarction size in the positive control group was considerable and comparable to similar studies in pigs [7]. Thus, the results obtained by coversin treatment appear coversin and not isoflurane mediated. Duration of treatment was relatively short with 4 h of reperfusion and conclusions about long-term myocardial complement activation, function and effect of coversin on LTB4 can therefore not be made. Thus, a pig closed-chest study with longer periods of treatment, reperfusion and observation should be performed prior to clinical trials investigating coversin in myocardial IRI [13, 40]. The trend to lower troponin-T and H-FABP levels during reperfusion in combination with reduced infarct size in coversin treated animals indicate that indeed myocardial IRI was reduced by coversin.

Pigs are regarded as one of the most translatable animal models in myocardial IRI research. Additionally, coversin has the same C5 binding characteristics in humans and pigs and coversin is already in clinical use in one eculizumab resistant patient as well as in phase Ib and II clinical trials (Clinicaltrials.gov NCT02591862 as well as producer’s webpage akaritx.com). Thus, the approach outlined in this study including the dosing regimen might be directly transferable to a clinical study investigating myocardial IRI when the long-term effects of coversin on myocardial cell survival and function as discussed above have been elucidated, complying with the proposed outline of future clinical studies targeting reperfusion injury in patients with myocardial infarction [16, 19].

In conclusion, we show in this clinically relevant model of myocardial IRI that complement inhibition of C5 reduces infarction size, possibly through reduction of IL-1β and E-selectin, and improves ventricular function. Accordingly, on the basis of concerns with previous studies and the results of this study we reason that there is a need to reconsider the use of complement inhibition especially at the level of C5 in clinical myocardial infarction.

## Electronic supplementary material

Below is the link to the electronic supplementary material.

Supplementary material 1 (PDF 93 kb)

Supplementary material 2 (PDF 279 kb)
