# Supplementary material for: Complement factor 5 blockade reduces porcine myocardial infarction size and improves immediate cardiac function
Source: Basic Res Cardiol. 2017 Mar 3;112(3):20. doi: 10.1007/s00395-017-0610-9 (PMC5336537; doi:10.1007/s00395-017-0610-9)
Supplement: Supplementary file 1 — Supplementary material 1 (PDF 93 kb) [file 395_2017_610_MOESM1_ESM.pdf]

Online Resource 1

Complement factor 5 blockade reduces porcine myocardial infarction size and improves immediate cardiac function

Basic Research in Cardiology

Pischke SE, Gustavsen A, Orrem HL, Egge KH, Courivaud F, Fontenelle H, Despont, A, Bongoni AK, Rieben R, Tønnessen TI, Nunn MA, Scott H, Skulstad H, Barratt-Due A, Mollnes TE.

Department of Immunology, Intervention Centre, Department of Anaesthesiology, Division of Emergencies and Critical Care, Oslo University Hospital, Oslo, Norway

s.e.pischke@medisin.uio.no

Supplemental table 1: detailed information about pigs (*Sus scrofa*) used in accord with ARRIVE guidelines [1]

Item	Explanation
Species and strain	*Sus scrofa*, NOROC
Weight	19.8 ±0.7 kg
Age	2 to 3 month old
Sex	11 males : 10 females
Origin	Open-air farm (Fredrikstad, Norway)
Health status	No signs of infections (no diarrhoea, no infected scratching marks)
Housing	Pig boxes (2.4 sq.m) with raised floors in a 40 sq.m room
Bedding	Aspen bedding (B&K Universal Ltd, Hull, UK)
Housing atmosphere	Tightly regulated room temperature (20°C) and humidity (55.7%), as well as 12 hours light/dark cycles.
Feeding	Unlimited access to commercial pig feed and tap water
Acclimatization period	One day and night
Transport to OR	Sedated by intramuscular injection of ketamine (800 mg), azaperone (80 mg), and atropine (1 mg) in the cage. An intravenous catheter was established in an ear vein and sedation deepened using pentobarbital (0.5 – 1 mg/kg).

OR atmosphere	Sterile and stable environment with regulated temperature (22°C) and humidity (50-60 %).
Temperature	Controlled with heating blankets to 38°C arterial temperature.
Anaesthesia	Balanced anaesthesia with Isoflurane 1.0-1.5% end tidal concentration and morphine 1-2 mg kg^{-1} h^{-1} infusion. Anaesthesia depth was regularly evaluated by no reaction to sharp hoof and nose pinching.
Respiration	Standard respirator (Leon, Heinen Lowenstein, Bad Ems, Germany). Tidal volume and respiratory rate adjusted to keep arterial pH at 7.36-7.44, arterial oxygen saturation > 94% and arterial oxygen tension > 10 kPa.
Hemodynamic monitoring	Central venous access via the external jugular vein. Artery catheters placed in the carotid artery for blood sampling and in the femoral artery for thermal dilution cardiac output measurements (PiCCO, Pulsion AG, Munich, Germany). Normovolemia was achieved by bolus infusion of 1000 ml Ringer acetate during the first two hours of the experiment to compensate for food deprivation and loss of sympathetic tone due to the induction of anaesthesia. Thereafter a background infusion of Ringer acetate (12 ml kg^{-1} h^{-1}) was given throughout the experiment.
Euthanasia	Bolus of Pentobarbital (300 mg), Morphine (10 mg) and Potassium chloride (50 mmol)

OR: operation room, iv: intravenous

References

1. Kilkenny C, Browne WJ, Cuthill IC, Emerson M, Altman DG (2010) Improving bioscience research reporting: the ARRIVE guidelines for reporting animal research. PLoS Biol. 8:e1000412 doi:10.1371/journal.pbio.1000412
