# Supplementary material for: Complement factor 5 blockade reduces porcine myocardial infarction size and improves immediate cardiac function
Source: Basic Res Cardiol. 2017 Mar 3;112(3):20. doi: 10.1007/s00395-017-0610-9 (PMC5336537; doi:10.1007/s00395-017-0610-9)
Supplement: Supplementary file 2 — Supplementary material 2 (PDF 279 kb) [file 395_2017_610_MOESM2_ESM.pdf]

Online Resource 2

Complement factor 5 blockade reduces porcine myocardial infarction size and improves immediate cardiac function

Basic Research in Cardiology

Pischke SE, Gustavsen A, Orrem HL, Egge KH, Courivaud F, Fontenelle H, Despont, A, Bongoni AK, Rieben R, Tønnessen TI, Nunn MA, Scott H, Skulstad H, Barratt-Due A, Mollnes TE.

Department of Immunology, Intervention Centre, Department of Anaesthesiology, Division of Emergencies and Critical Care, Oslo University Hospital, Oslo, Norway

s.e.pischke@medisin.uio.no

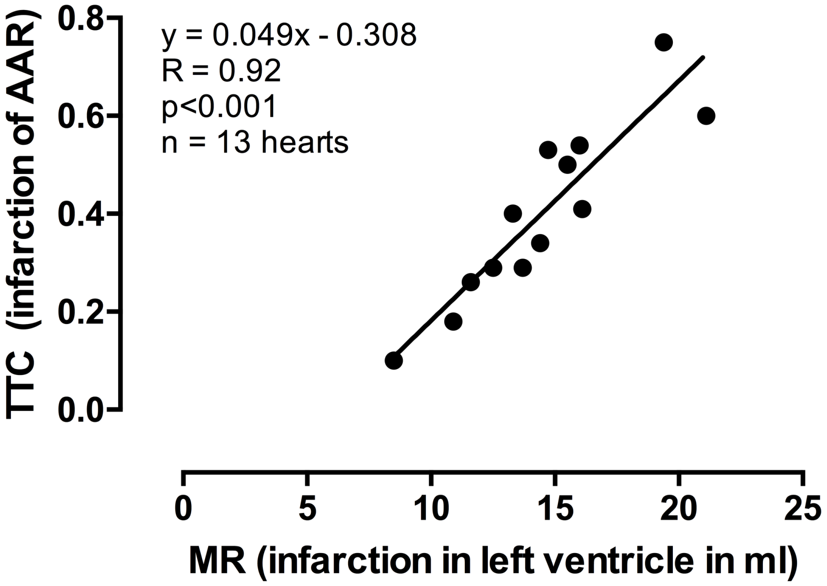

Pearson correlation analysis was performed between infarct sizes determined by tetrazolium chloride (TTC) staining using the infarcted percentage of the area at risk (AAR) and by magnetic resonance (MR) determined infarction size of the left ventricle in ml.
